# Planning for implementation and sustainability of a community-based suicide surveillance system in a Native American community

**DOI:** 10.1186/s43058-022-00376-1

**Published:** 2023-01-04

**Authors:** Teresa Brockie, Ellie Decker, Allison Barlow, Mary Cwik, Adriann Ricker, Theresa Aguilar, Lawrence Wetsit, Deborah Wilson, Emily E. Haroz

**Affiliations:** 1 Johns Hopkins School of Nursing, 525 North Wolfe St, Baltimore, MD 21205 USA; 2 Johns Hopkins Bloomberg School of Public Health, Department of Mental Health, 615 N. Wolfe St, Baltimore, MD 21205 USA; 3 Johns Hopkins Bloomberg School of Public Health, Center for Indigenous Health, 415 North Washington St., 4th Floor, Baltimore, MD 21231 USA

**Keywords:** Sustainability, Community and cultural asset mapping, Suicide prevention, Community-based participatory research, Native American, Surveillance

## Abstract

**Background:**

Native American youth, primarily living on reservations, suffer the highest burden of suicide of any racial group in the USA. Implementation and sustainability of culturally grounded, evidence-based interventions are needed to address suicide in Native American populations. For nearly 40 years, Montana has ranked at or near the top nationwide for suicide. Fort Peck Tribal leadership declared a state of emergency in 2010 after six suicides and 20 attempts that occurred over a 5-month period.

**Methods:**

We used a community-based participatory research approach for adapting the Celebrating Life (CL) program with a specific focus on long-term sustainability, which has demonstrated efficacy in addressing suicide with the White Mountain Apache. The aims were to (1) adapt the CL program intake forms through roundtable discussions, (2) conduct asset and resource mapping to identify community and cultural resources to leverage for the CL program within the Fort Peck context, and (3) develop a sustainability plan for CL in Fort Peck through qualitative approaches informed by the Program Sustainability Assessment Tool.

**Results:**

Roundtable discussions resulted in adapted intake forms that capture variables relevant to the Fort Peck context. Asset mapping identified 13 community assets and 10 cultural resources to incorporate within the CL implementation process. Focus group discussions yielded four key themes that were incorporated into a plan for sustainability: (1) strategic partnerships, (2) long-term funding, (3) communication planning, and (4) workforce planning and engagement.

**Conclusions:**

This paper outlines an avenue for using culturally adapted tools to design an implementation system driven by community and cultural assets within tribal communities and for integrating program planning for sustainability early in the implementation process.

**Supplementary Information:**

The online version contains supplementary material available at 10.1186/s43058-022-00376-1.

Contributions to the literature

- We utilized community-based participatory research methods and a strength-based approach to adapt a suicide surveillance program for implementation.
- The methods described provide a process for planning prior to implementation to increase acceptability, sustainability and engagement with community assets, factors shown to be critical for long-term program sustainability.
- Findings demonstrate the importance of cultural and contextual adaptations prior to program implementation.

## Background

Native American (NA) (American Indian/Alaska Native) youth living on reservations and in rural settings suffer the highest burden of suicide of any racial group in the United States (US) [1]. Key risk factors comprise various mental health problems including depression, trauma, substance use, impulsivity, loss of cultural identity, low self-esteem, and hopelessness [2]. Health system-level barriers, including scarcity of mental health services and providers—particularly NA providers, a lack of tribe-specific data to inform intervention development, and limited resources for long-term funding stability negatively impact the ability to address NA suicide rates [3]. However, NAs’ cultural understanding of mental health, culturally informed protective factors, and a preference for culturally based healing modalities are under-developed assets in the context of suicide [4]. More specifically, the unique social structures and interconnectedness that exist among NA families, social systems, and extended networks provide a safety net that can foster protective factors including positive social support, connection to community, identity, and supportive cultural practices [5]. A culturally grounded approach that aims to reduce risk and foster protective factors, while focusing on community and cultural assets and simultaneous planning for long-term sustainability, holds promise for reducing NA suicide.

Research and programs implemented in NA communities experience the common challenge of long-term sustainability. It is a familiar and persistent problem that promising programs wither when funding ends, which is demoralizing for tribal communities [6]. While implementation and sustainability of culturally grounded, evidence-based interventions are needed to address suicide in NA populations, there has been little research focused on adaptation, implementation, and sustainability of programs proven effective in other NA communities [4]. More often, programs developed and tested in non-NA populations are implemented but are not adapted or are poorly adapted [7]. This process requires significant work to ensure cultural and contextual relevance, while maintaining the effectiveness of the original intervention—a resource-intensive process that is not always achievable [8]. Even with programs developed or significantly adapted and tested in NA communities, many programs struggle to sustain or scale after the grant-funded period due to systematic underfunding of the health systems that support such programs (e.g., Indian Health Service (IHS)) and the persistent incongruence between US health services and NA values and practices [9]. Meanwhile, longstanding, multi-generational social determinants such as high unemployment, concentrated poverty, poor access to physical and mental health care, historical trauma, and systemic racism continue to drive up suicide risk and elevate the need for sustained evidence-based programming [10, 11].

Assessing sustainability capacity, or the ability to maintain programming and its benefits over time, should be an integral step in research and program implementation as evidence-based programs in virtually all settings are challenged with sustaining activities over time. Understanding factors that contribute to long-term viability can prevent early closure of crucial public health programs, particularly in low-resource settings [12]. The Program Sustainability Assessment Tool (PSAT) has been used to inform sustainability planning for such programs. Planning for suicide surveillance and case management sustainability in a low-resourced tribal community prompted community engagement to understand factors related to suicide prevention and long-term sustainability in this context.

Asset mapping, which is the process of collecting, recording, analyzing, and synthesizing community and cultural resources, and networks supports capacity-focused development, is in direct contrast to “needs assessments,” which are often deficit-oriented. Further, deficit-oriented approaches often exclude communities from active roles, thus perpetuating structural inequities and failing to secure lasting change [13]. Community asset mapping has been successfully utilized with First Nations communities in Canada and Māori in New Zealand to understand behaviors and develop feasible and sustainable interventions in low-resource settings related to healthy food access and maternal health care [14, 15]. Cultural asset mapping primarily occurs through collective discussion and strategic collaboration, which requires intentional self-reflection and sharing to identify pertinent cultural assets for developing tailored solutions to a problem. This process was used to engage the communities in self-reflection to improve the quality of suicide prevention strategies while respecting tribal sovereignty and autonomy [4, 16, 17].

In response to a suicide cluster in 2001, the White Mountain Apache Tribe, in collaboration with the Johns Hopkins Center for Indigenous Health (CIH), developed the *Celebrating Life Suicide Surveillance and Case Management System*. Celebrating Life (CL) involves implementation of a reservation-wide, tribal-mandated reporting system that prompts assignment to a case manager and appropriate referrals, and longitudinal follow-up. Over the past decade using six caseworkers and a supervisor, CL has shown increases in (a) reports to the system over time, demonstrating greater community awareness of the system and self-efficacy about how to report; (b) the ability to locate people at increased risk, indicating greater skill among the CL team to work with individuals on a sensitive, sometimes stigmatized issue; and (c) referrals to mental health services and improved linkages to care. Over the past 20 years, CL has provided services to over 2000 individuals and yielded decreases in suicide attempts and death rates over time. During the first 6 years that the CL surveillance and paired case management system was implemented, the all-ages Apache suicide rate decreased by 38.3%, and the rate for 15–24 years old decreased by 23%. Additionally, the annual number of attempts decreased from 75 to 35 in the years 2007–2012 [16]. The CL program is a prime example of a sustainable and evidence-based suicide intervention that is now being disseminated to other tribal communities through a National Institute of Mental Health collaborative hub mechanism (5U19MH113136-02).

For nearly 40 years, Montana has ranked at or near the top nationwide for suicide, with a crude rate of 23.8 deaths per 100,000 in 2014 [18]. In 2010, Fort Peck (Montana) Tribal leadership declared a state of emergency after six suicides and 20 attempts occurred over a 5-month period. That same year a collaborative partnership was established between the Fort Peck Tribes and the lead author, a NA nurse researcher that led to a cross section correlational design study exploring risk and protective factors for youth suicide. The study found that among 288 Fort Peck Assiniboine and Sioux youth 15–24 years of age, 35% reported suicide attempts, and 45% reported suicide ideation [19]. Further, suicide ideation and attempts were associated with family history of negative mandatory boarding school experiences (AOR=4.8) and poly-drug use (AOR=3.6). Suicide attempts were also associated with post-traumatic stress disorder (AOR=2.6) and depressive symptoms (AOR=3.6) [19]. The isolation of living in a remote area coupled with local and regional underfunding of the IHS are obstacles to timely and appropriate mental health services [20, 21], leaving individuals more vulnerable to suicide [22].

**Fig. 1:**
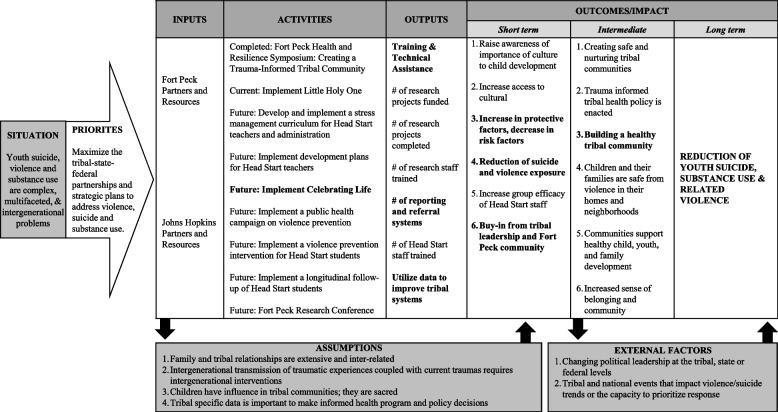
Fort Peck Logic Model. Bolded items show anticipated outputs and outcomes/impact as a result of CL implementation

Since 2010, the collaborative partnership has expanded to include five Assiniboine and Sioux onsite research team members and evolved to include other research and activities. Figure 1 is our Logic Model of complete, current, and future activities; outputs; and outcomes through our collaborative work with Fort Peck Tribes. This model is updated annually by our Tribal Advisory Boards (TAB) and community partners. Our current efforts include a randomized control trial *Little Holy One* and implementing *Celebrating Life*. Importantly, our collective efforts have remained the same, the long-term goal to reduce youth suicide, substance use, and related violence. The model highlights the importance of multi-level interventions to increase protective factors and decrease risk factors related to youth suicide. Our work is guided by a TAB [23, 24], comprised of Assiniboine and Sioux members who work in systems of care for individuals with suicide risk: they direct our efforts and ensure alignment with Fort Peck needs and culture [23].

Adversity is common among reservation-based NA youth, as many face factors that place them at increased risk for morbidity and mortality including poverty, social isolation, fractured families, and adverse childhood experiences [10, 11]. Additionally, mental health services in this isolated, rural reservation are limited, and preventive mental health services, especially for adolescents, are beyond the tribes’ limited resources. IHS is primarily responsible for health care delivery for Federally Recognized Tribes; however, under Public Law 93-638, tribes or tribal organizations may opt to administer programs or services, such as behavioral health services [25]. The Fort Peck Tribes provide several services through a 638 contract including substance use treatment through the Spotted Bull Resource and Recovery Center and youth behavioral health services available in schools, although not mental health services. The Fort Peck IHS Unit includes the Verne E. Gibbs Health Center located in Poplar and the Chief Redstone Health Clinic located in Wolf Point, which provide all outpatient care on the reservation. Two practitioners, a psychologist and licensed clinical professional counselor provide all behavioral health services. There are no services for inpatient psychiatric care; any acute psychiatric needs are addressed in-state (up to 460 miles from Poplar) or out-of-state (199 miles from Poplar at Trinity Hospital in Minot, North Dakota). Adolescent psychiatric needs are addressed in Billings, Helena, or Minot. Long-term psychiatric patients are referred to the Montana State Hospital in Warm Springs (502 miles from Poplar); however, because IHS only allocates financial provisions for acute mental healthcare, patients are required to pay for services. There are few sustained efforts for addressing community mental health needs, especially those that focus on suicide prevention in remote, low-resource communities [22]. Difficulties accessing and receiving needed health care increases vulnerability to suicide [22]. Accordingly, the TAB chose to adopt the White Mountain Apache CL program to address the high prevalence of suicide on the Fort Peck Reservation.

Developing public health programs to address suicide and related mental health challenges in NA communities requires careful consideration of the local cultural and implementation context and a proactive plan for sustainability [26]. To be most successful, local communities need to be engaged in the process of understanding factors that promote and hinder the sustainability of these programs [27, 28]. Thus, the selection of *Celebrating Life* and the inclusion of asset mapping and sustainability planning was informed by the TAB. Community and cultural asset mapping, focused on inventorying the resources and strengths of a community, can be a useful approach to assist with revealing solutions to the most persistent implementation and sustainability challenges [29]. Recognition of the inherent assets within NA worldviews and values and leveraging them as the basis for public health prevention programming can inform a strengths-based approach to suicide prevention in this context.

The objective of this paper is to describe the process of adaptation, identification of community and cultural assets, and sustainability planning for the CL Suicide Surveillance Program in the Fort Peck Reservation context [17]. The TAB directed the formative phase of the project and will assist in interpreting and applying findings. This study had three aims: (1) roundtable discussions to adapt current intake and case management forms; (2) roundtable discussions using the adapted University of California, Los Angeles (UCLA) Asset Mapping guide to map community assets and resources for adapting and implementing the suicide surveillance and case management system within existing tribal systems, processes, and resources; and (3) focus group discussions (FGDs) using the adapted PSAT to inform a sustainability plan. Community-based participatory research (CBPR) utilizes academic community partnerships to develop collaborative interventions to address public health concerns identified by the communities and engage community members [24]. The CBPR approach used in this work supports both tribal ownership and reflection on community assets.

## Methods

In this qualitative study, we used a CBPR approach to conduct roundtable discussion sessions and three FGDs to adapt CL intake forms and to integrate the perspective of the community and service providers for asset mapping and sustainability planning. These processes were conducted in preparation for the implementation of CL, which we are now seeking funding support. Onsite research team members AD and LW conducted the roundtable and FGDs. Both are from the community and experienced in qualitative data collection. All sessions were recorded and transcribed verbatim. As described above, the project is overseen by a TAB that meets quarterly and as needed during active phases of the project.

### Setting and sample

The 2-million-acre Fort Peck Reservation, established by treaty in 1888, is home to more than 13,000 enrolled Assiniboine and Sioux members. Located in northeastern Montana, it is extremely remote, with a population density of five people per square mile—significantly less than the US average of 90 people per square mile. There are six distinct communities within the reservation’s external boundaries: Poplar, Wolf Point, Frazer, Oswego, Brockton, and Fort Kipp. The population on the reservation is primarily made up of young residents, with a median age of 27.9 years, compared to Montana’s median age of 39.8 years.

### Participants

We purposively sampled 13 Assiniboine and Sioux community members, including seven members of our 9-person TAB, to participate in CL form adaptation, community and cultural asset mapping, and sustainability planning. Recruitment was by invitation only because participants were required to be knowledgeable about Assiniboine and Sioux culture and/or to have worked in systems of care for individuals with suicide risk. Participants included four females, nine males; two spiritual leaders, one from each tribe; a behavioral health provider; suicide crisis response team member; a family member who had lost a child to suicide; and representatives from Spotted Bull Recovery Resource Center, IHS, Tribal Health, Fort Peck Tribal College, and the State of Montana. Each participant had experienced either a family member, close relative, or friend who had died by suicide.

### Ethical considerations

In 2017, Tribal leadership authorized this research project through Tribal Resolution #29-15502017-1, which includes a provision for protection of Indigenous knowledge and ensures accurate representation. The Johns Hopkins School of Medicine Institutional Review Board (IRB) and the Fort Peck Tribal IRB provided human subjects approval. All participants completed written consent prior to the start of activities so the results of our project could be documented and shared. Finally, the Fort Peck IRB reviewed and approved this manuscript for publication.

### Celebrating life surveillance and case management system

Paramount to the success of White Mountain’s CL surveillance and case management is their tribal law that mandates reporting of all suicide-related events within tribal jurisdiction [30]. The law directs all persons, departments, and schools within tribal jurisdiction to report, via a standard intake form, any observed or documented suicide ideation, attempts, deaths, and binge substance use to a centralized task force of Community Mental Health Workers managed by the CIH [16, 31]. The intake form collects demographic information (age, gender, residential, and marital status), type and location of the event, and the reporter’s name. Intake forms received by the centralized registry are managed by a local Apache CL team and then routed to an assigned case manager. The case manager seeks out the reported individual to conduct an in-person interview where they (1) complete a case management form to validate the event, (2) collect additional risk and protective factor data (i.e., education and/or employment status, history of other suicide-related behavior, recent losses of family or friends to suicide or other violent death, reason for act, and use of substances during act), and (3) assess the individual’s imminent risk. The interview occurs at the person’s home, school, or another private setting. Together, the CL team and CIH review the information and determine the coding (confirmation) for the event, and case managers follow them for 30–90 days depending on their risk category. The CL team makes appropriate referrals and plans for “wellness checks” for those at imminent risk to ensure their safety between referral and appointment time and, when possible, provides a warm hand-off. Consistent with brief contact intervention approaches, case managers conduct wellness checks on an ongoing basis as indicated by clinical judgment and/or per individual requests. All data from the intake and case management forms are entered into a secure, web-based database. This innovative public policy approach to suicide prevention demonstrates the importance of tribal sovereignty and governance as a tool to affect change.

### Data collection

#### Form adaptation

The CL intake and case management forms were reviewed and revised through a half-day roundtable discussion with (*N*=5) participants, two local (AR, LW) and three visiting study team members (TB, DW, ED), to make necessary adaptations for the Fort Peck context. First, the research team described the purpose of the roundtable discussion and then provided an overview of the CL Suicide Surveillance Program, including how the forms were utilized in the White Mountain Apache context. Participants were then provided a copy of each form and asked to review on their own, with a keen focus on CL implementation at Fort Peck. The group then came together to discuss suggested adaptations. Any disagreements were resolved by culturally appropriate consensus decision-making.

#### Asset mapping

We first adapted the six-step UCLA Asset Mapping guide to include a step focused on identifying cultural resources for addressing suicide. As such, the seven steps were as follows: (1) defining community boundaries; (2) identifying and involving partners; (3) determining what type of assets and resources are available; (4) determining what cultural assets are available (added step); (5) listing assets (organizations, associations, and institutions in the community); (6) listing assets of individuals in the community; and (7) organizing a visual map or diagram about where these assets (resources, organizations, and individuals) are and how they communicate with each other. To explore these domains, we conducted a roundtable discussion with participants (*N*=4), which included both discussion and individual written responses to asset mapping questions. To facilitate discussion, we developed a guide (available upon request) comprised of eight questions with multiple prompts based on the UCLA Asset Mapping guide. When completing individual responses, participants were asked to identify the most important assets and factors and then rank them in order of importance. To organize a visual map, each participant was provided a map of Fort Peck to identify assets across the reservation. Next, participants’ individual maps were reviewed and compiled on a large map for collaborative viewing. Finally, they verified results and added any missing resources. At each step, participants were reminded of the primary purpose of the activity to re-center focus around suicide prevention in the community. Written and oral responses were compiled by each domain, and the discussion was audio recorded and transcribed.

#### Sustainability planning

The PSAT is based on the *Sustainability Framework* developed by the Center for Public Health Systems Science at Washington University in St. Louis [28]. The 40-item questionnaire measures capacity for sustainability through items across eight domains. The tool is designed to inform action plan development to increase likelihood of program maintenance and benefits longitudinally. While the PSAT is typically administered as an individual questionnaire designed for existing programs, we adapted the PSAT into a qualitative interview guide (available upon request), to facilitate discussion on the development of a long-term sustainability plan prior to implementation. The inclusion of sustainability planning in the development process was intuitively embraced by participants and local research staff, signifying a welcome break from living hand-to-mouth on grants without a vision for long-term sustainability of successful programs.

**Fig. 2:**
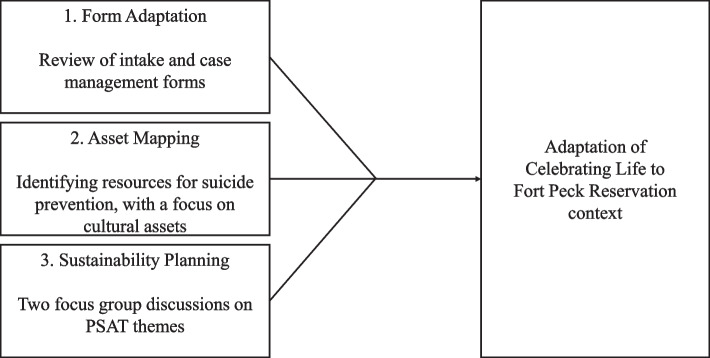
Research study process flow

We conducted two FGDs with six and five community members, respectively, who currently work or have previously worked in systems of care for individuals with suicide risk. Recruitment was conducted using email invitations and word of mouth. Participants were provided with an overview of CL and the purpose of the FGD. They were then asked to consider CL implementation on Fort Peck based upon their experience(s) with suicide prevention programs. The adapted guide included nine questions and prompts that focused on six of the nine PSAT domains: (1) environmental support, (2) funding stability, (3) partnerships, (4) organizational capacity, (5) program evaluation, and (6) communications. We excluded the program adaptation and strategic planning domains, as we plan to address these domains during the initial planning phase of implementation. Additionally, the environmental support domain was adapted to include tribal support. An additional staff member (DW or ED) was present to manage logistics and take notes on participants’ responses, body language, and engagement. Overall research study flow is outlined in Fig. 2.

### Data analysis

Analysis procedures were developed for each of the three data collection activities. For the first two parts (form adaptation and asset mapping), participants’ responses were compiled and reviewed for consensus by the TAB and study team. A framework analysis approach was utilized for part three (sustainability planning) to sort and code focus group discussion question responses by the six domains described above using Microsoft Excel. Quotes and emerging themes were recorded in the relevant framework cells. For example, in response to the question “What types of funding have previous successful programs utilized?,” comments were categorized in the “funding stability, short term” category. Coding was performed by two team members (AR, TA) who met weekly to address coding discrepancies and reach consensus. AR is a member of the Fort Peck community and resides on the reservation. TA is external to the Fort Peck community and trained in qualitative data analysis. The consolidated criteria for reporting qualitative research (COREQ) was utilized as a qualitative reporting guideline and has been included as Additional file 1 [32].

## Results

### Form adaptations

Recommended adaptations focused on form content (e.g., adding a question or response option) and structural changes were not raised. Recommended adaptations (see Table 1) were made to both the intake and case management forms and will undergo final review by the TAB and study staff prior to implementation. Original intake and case management forms from the White Mountain Apache Tribes Celebrating Life Suicide Surveillance and Case management system can be found at Cwik et al. [31]. Specifically, additional questions about drug use and previous trauma were added for clarity. Questions were also added to assess access to housing and traditional ceremonies.

**Table 1:** Recommended changes to intake and case management forms

Intake form	Case management form
Add question to assess current living arrangements/housing status	Add question: “Do you attend traditional ceremonies?”
Gender: Add answer choice of “Other: ________”	Add question to learn how client prefers to get information about suicide prevention and resources
Add question to learn if client is a parent or caregiver	Add answer choice of “meth” to method(s) used options
Education: Add school client attends	Add question to learn if there is a peer or mentor the person feels comfortable talking with. Collect name and contact information of that person.
Type of self-harming behavior: Add answer choice of “opioid use”, “experimental drug use”, “substance use”	What was the reason for your act: Add answer choices of: “past experiences of trauma (unreported)”, “past experiences of inflicting trauma or pain on someone else”, “guilt from previous choices”, “meth”, “break-up”
Method: Add answer choice of “train”, remove answer choice of “jump”	Add question: “How are you administering these drugs?” with answer choices: “pills”, “injection”, and “smoking”
Add question to learn if there was a triggering event preceding the attempt	Add question “Did you blackout?” to section assessing binge substance use
Add name of person who made report. Add question about how the reporter learned about CL	Modify referral agencies listed to Fort Peck Reservation services
	Add questions to assess past meth and inhalant use
	Add question: “Do you know anyone who has attempted suicide in your lifetime?” If yes, “How many people?” and “What was the relationship?”
	Add prompts of: “How many people?” and “What was the relationship?” to the question “Do you know anyone who has died by suicide in your lifetime?”
	Add question: “Have you lost a loved one in the past 6 months?”
	Change question from “Have you ever experienced domestic violence (verbal or physical abuse)? to “Have you ever been hit, kicked, slapped, or punched?”
	Add question: “Have you ever been the perpetrator of abuse?”
	Add question: “Have you ever seen your parent be slapped, hit, kicked, or punched? If yes, how many times?”
	Add questions about sexual activity, consent, rape, and miscarriage

### Community and cultural assets

During asset mapping, there was in-depth discussion among participants regarding important community and cultural assets which may positively influence or hinder successful suicide prevention program implementation in the Fort Peck community. In total, participants identified 13 community assets and 10 cultural assets (Table 2). When asked, “which community assets are most utilized to address suicide,” a variety of organizations were listed, including federal, tribal, and state. Additionally, two cultural assets “traditional individuals (Lodge Family, Sundance)” and “natural helpers” were included, indicating participants were in tune with cultural assets. Similarly, when asked, “What cultural assets are most utilized to address suicide?,” responses ranged from “ceremony” to “elders who know traditions and language.” When further asked to list factors important for program success, participants’ responses included clear and direct communication, need for interagency coordination, and financial resources.

**Table 2:** List of community and cultural assets to address suicide identified by TAB through asset mapping

**Community assets**	
IHS Behavioral Health	
Health Promotion Disease Prevention (HPDP)	
Montana Office of Public Instruction - System of Care Tribal Wraparound Project	
Traditional Individuals (Lodge Family, Sundance)	
Natural Helpers	
Crisis Response Team	
Youth Council	
Spotted Bull Resource and Recovery Center	
Northeastern Montana Health Services	
Families/organic systems	
Law Enforcement	
Red Bird Woman Center	
Tribal Courts	
**Cultural assets**	
People who are traditional and open to helping others develop their cultural knowledge	
Creator’s Game	
Medicine Wheel	
Language Department at Fort Peck Tribal College	
Ceremony (Sundance, Sweat Lodge, Medicine Lodge)	
Resilience of tribal people	
Elders who know language and traditions	
Lessons that can be applied to life situations	

### Program sustainability themes

Key themes related to programmatic sustainability for the six PSAT domains identified by participants are highlighted below.

#### Tribal and environmental support

Key themes identified were consistent engagement with the community, including partnering with active community leaders, Tribal Council involvement to promote cooperation across organizations, and avoidance of inter- and intra-departmental conflicts. Participants discussed challenges that arise when organizations had limited understanding of protocols and capabilities of partner organizations. For example, one participant highlighted that each school on the reservation has different protocols for reporting suicidal behavior. FGD participants also identified key topic areas to address locally, such as revision of the tribe’s misdemeanor law for suicide risk and support of a tribal resolution that mandates reporting, similar to the White Mountain Apache model.

#### Funding stability

Participants noted that non-grant funding was critical to program sustainability for many reasons—specifically staffing and billable services. Participants described staffing challenges related to stress, burnout, and antagonistic relationships, as well as more infrastructure-related challenges such as limited orientation procedures and unfair pay differential between organizations. Potential solutions focused on employee wellness and incentives, building a supportive teamwork environment across organizations and departments, and bolstering programs’ autonomy. Regarding billing, third-party billable services and 638 contract monies were described as key funding sources for programs avoiding over-reliance on grant funds.

#### Partnerships

Important partnerships identified at the local level were the county health department, Sheriff Department, parents, the Tribal Executive Board, and teachers. Lack of clarity regarding partnership schedules, services, roles, procedures, and capacities were reported challenges. One participant described a circumstance whereby a lack of clarity resulted in a yearlong project delay, underscoring the importance of establishing clear goals and expectations upfront. Participants noted that several partnerships had been established across reservation organizations and partners met regularly. Even so, one participant noted:

I feel like we have a good amount of partnerships and when they have failed, it’s because we have different expectations of time and services delivery. … And I think it would be easy, like if there’s going to be community asset mapping, it would be nice to know [organization] can be partnered in these works and [organization] partners really well in these areas…. (FGD 2 participant)

When asked to describe how partnerships should be built and facilitated for the implementation of CL, participants suggested beginning with a clear understanding of policies, procedures, and capabilities of *all* partners and establishing a formal partnership, through a memorandum of agreement, between all organizations. Participants stated that a clear action plan, with tasks and deadlines assigned to specific individuals and organizations, would be critical to successful implementation of CL, with clarity of capabilities, policies, and goals necessary for successful partnerships.

#### Organizational capacity

Participants described the importance of an organization’s capacity to engage with the community and instill a value system of integrity and trust with its employees. Lacking community trust hinders organizations’ ability to deliver their product or service. Participants described ineffective and unclear organizational processes as a reason for lack of community trust:

…I don’t trust the reporting system. … There is the issue, okay, why do I not have trust in the social services department, and if I call social services, what’s going to happen to this kid and is it going to be better or worse than what’s already happening to them? (FGD 2 participant)

Further noted challenges included staffing shortages, undefined roles, competing tasks, and the importance of confidentiality and professionalism. Innovative reward systems, focused on culture and community, were utilized to build organizational capacity. One participant noted,

It’s a challenge to build in a reward system…There are no bonus opportunities. You have to be able to motivate your employees or coworkers, or team members. The one way we found that’s really helpful is going back to our traditional values and we start honoring them in a traditional way to say, okay, thank you for all your hard work. We’re going to recognize you in front of everybody because this is all we can do. (FGD 1 participant)

Participants felt that including traditional ways of honoring people could be incorporated into CL to aid in staffing and program sustainability.

#### Program evaluation

In this domain, participants were prompted to discuss how similar suicide prevention/response programs had been evaluated in the past and how results were utilized. The evaluation procedures described were initiated at the end of the grant to meet funding requirements, so external consultants often completed the evaluations. Some participants used quarterly evaluations to provide more immediate feedback and an ability to make changes more routinely. Regular evaluations provided an opportunity for teams to reflect and find motivation based on programmatic impact by quarter. However, the time-intensive process of quarterly data gathering was reported to be a barrier. Regular strategic planning and goal tracking was also described. Some participants utilized a tracking system that alerted employees about monthly and yearly goals and provided space for progress reporting; others tracked weekly tasks and hours to note both how time was spent and if key objectives were missed. When discussing future evaluations of the CL program, participants recommended starting with comprehensive baseline data of suicide attempts and completions before program implementation. CL program evaluation would focus on changes in numbers of suicide attempts and completions, number of clients who had accessed services, and qualitative feedback from clients.

#### Communications

Several communication strategies to secure and maintain public support were identified, including community activities like powwows, social media, and door knocking; engaging parents and those with high visibility; and leveraging spiritual leaders and bundle carriers by having them speak to the community about CL would increase the community’s willingness to utilize it, e.g., getting parents to give consent. One participant said, “We’re giving this underlying message […] but we are doing it in a manner that is exciting to youth. And then we are, really focusing on not just the individual, but families as a whole” (FGD 1 participant)*.*

This response emphasizes the importance of active engagement with the community and families. Participant suggestions to include youth included leveraging the Tribal Youth Council and hiring recent master’s graduates who have expressed interest in this work. They will be an important connection to youth and are comfortable with social media communications, which will bolster engagement.

## Discussion

We used a participatory, asset-based, and community-engaged research approach for adapting and planning for the long-term sustainability of an evidence-supported suicide prevention program. The three aims focused on specific programmatic adaptations and considered larger cultural and contextual factors that will support successful implementation and sustainability of effective prevention programming. Several themes emerged that will guide the implementation of CL and case management at Fort Peck. The planning work presented here was unique in that it takes a strength-based approach, by exploring both community and cultural assets, and considering long-term sustainability from inception. While the PSAT tool was initially created to evaluate sustainability of existing programs, our process speaks more to feasibility and long-term sustainability prior to program implementation.

Our initial adaptation process began with revising the intake and case management forms, which was helpful for familiarizing participants with the CL program and engaging them to gather their unique input about what factors are most important to collect from the surveillance system. Suggested changes resulted in a more extensive intake form. Moving forward, we will monitor if this results in data that are more comprehensive or presents a barrier to rapid reporting. Asset mapping familiarized the TAB members even further with the program and helped garner community engagement and motivation; previous strengths-based research studies have also reported benefits to community engagement [14, 33]. The benefits of utilizing asset-based approaches have been exemplified in multiple studies with Indigenous populations [14, 15]. Further, the importance of community buy-in and cultural adaptation described in the results is supported by Haroz et al. [34] research on factors needed to sustain suicide prevention programs in NA populations. Finally, use of the PSAT targeted key goals and priorities for ensuring sustainability, like (1) tapping into tribal and environmental capacity for reducing employee turnover and mitigating job stress, (2) leveraging long-term funding stability through 638 contract monies and third-party billing, (3) targeting specific partnerships that could support CL and enhance the Tribal Suicide Prevention Plan, (4) using communication strategies that enhance visibility in the community and via social media, and (5) dovetailing suicide prevention with substance abuse prevention, which the White Mountain Apache Tribe has also identified as a critical approach.

The inclusion of sustainability planning in the development process was a crucial step often forgotten in planning for implementation of evidence-supported programming. Prospectively planning for sustainability if done is often done during implementation of programs [35]. However, pre-implementation is an even more critical time for considering factors that may enhance the capacity of a program to sustain itself beyond the funding period, particularly in this context. Funding is only one aspect of sustainability; building and enhancing other components that lead toward long-term sustainment of high-quality programing is critical to ensure continued impact of evidence-based programs. Important next steps are to line up sustainable resources for implementation, hire and train local staff, and conduct ongoing monitoring and evaluation. The 638 contract monies refer to Public Law 93-638, the Indian Self-Determination and Education Assistance Act, which allows tribes to transfer funds and responsibilities for a federal program, function, service, or activity from the federal government to the tribe. For example, under Public Law 93-638, the tribes could, through a government-to-government relationship with IHS, take control of health care programs provided by IHS [30, 36, 37].

In the White Mountain Apache community, maintaining and sustaining CL has succeeded due to continuous renewal and expansion of the tribal mandate; a strong network of health and human service providers, tribal members, respecting and supporting the mandate; ongoing monitoring of the system to demonstrate its impact; and public and private funding that has been attracted to the ongoing need and success of the system [16, 31]. It will be important to track if this pathway or others are shared by Fort Peck and other tribes across the country adopting the CL suicide surveillance and follow-up systems.

## Conclusions

In the end, participants and the TAB identified three critical next steps for implementing and sustaining CL at Fort Peck: (1) addressing the Tribal misdemeanor law for suicide risk; (2) implementing a tribal resolution that mandates reporting suicide-related behavior within tribal jurisdiction, similar to the White Mountain Apache model; and (3) exploring program sustainability through third party billing. Our findings have some important limitations. First, sample size was small due to inclusion criteria requiring experience with systems of care for suicide. This criteria prevented input from the wider community and youth at this pre-implementation stage. Further, participants in underserved populations are often difficult to reach due to remoteness and distrust [38]. Thus, we may have missed some valuable insights into program sustainability. Second, our study is limited to a two-tribe one-reservation sample and may not generalize to other NA settings. However, it should be noted that this reservation is like many isolated, rural reservations with similar socio-economic profiles.

Community and cultural asset-focused program development strategies engage local strengths to promote effective community interventions that last. Suicide is a complex problem, and even more so in remote NA communities that have experienced decades of fragmented, under-resourced, and culturally incongruent systems of care that, like NA suicide inequities, are the direct result of colonization and its aftermath. The aforementioned development process for implementing and sustaining the CL system can be used as a model by other tribes for their own unique public health programming. If tribes are not positioned to conduct this resource-intensive process, the key themes from these efforts may be considered when adopting CL or other similar programs. This CBPR study serves as an exemplar for adapting, building, and sustaining culturally congruent interventions in ways that empower historically marginalized communities.

## Supplementary Information

**Additional file 1.** COREQ.

## Data Availability

The government-to-government relationship between the US and federally recognized tribes acknowledges tribal sovereignty, tribal laws, and the tribes’ capacity to make decisions about research that is conducted within their jurisdictional boundaries. The tribal law that supports this research acknowledges the importance of community consent as well as individual consent and will provide the particularities for data sharing as part of tribal research regulation. All resources and data generated by this project will be made available in the most user-friendly, accessible, secure, and ethical format to all study partners. We will take necessary steps to ensure we adhere to Fort Peck Tribal Law (Resolution #28-1744-2017) and National Institutes of Health guidelines on sharing of data, in collaboration with our tribal partners, including seeking the appropriate tribal approvals to respect their tribal sovereignty and confidentiality.

## Abbreviations

NA(s): Native American(s)
US: United States
CL: Celebrating Life
PSAT: Program Sustainability Assessment Tool
TAB: Tribal Advisory Board
IHS: Indian Health Service
IRB: Institutional Review Board
CIH: Center for Indigenous Health
COREQ: Consolidated Criteria for Reporting Qualitative Research
HPDP: Health Promotion Disease Prevention
MOA: Memorandum of Agreement
