# Supplementary material for: Planning for implementation and sustainability of a community-based suicide surveillance system in a Native American community
Source: Implement Sci Commun. 2023 Jan 4;4:1. doi: 10.1186/s43058-022-00376-1 (PMC9814428; doi:10.1186/s43058-022-00376-1)
Supplement: Supplementary file 1 — Additional file 1. COREQ. [file 43058_2022_376_MOESM1_ESM.docx]

| **Item** | **Response** | **Page** |
| --- | --- | --- |
| **Domain 1: Research Team and Reflexivity** | | |
| *Personal Characteristics* | | |
| 1. Interviewer/facilitator | Lawrence Wetsit led focus group discussion (FGD) 1, with assistance from Teresa Brockie. Adriann Ricker led FGD 2, with assistance from Teresa Brockie. Ellie Decker provided logistical support for both FGDs. Round table discussions for asset mapping and form adaptation were led by Teresa Brockie, with assistance from Ellie Decker and Deborah Wilson. | N/A |
| 2. Credentials | Teresa Brockie, PhD, MSN, RN Ellie Decker, MSPH Adriann Ricker, MPH Theresa Aguilar, MD, MPH Lawrence Wetsit, MBA Mary Cwik, PhD  Deborah Wilson MPH, MSN, RN Allison Barlow, PhD, MPH Emily Haroz, PhD | N/A |
| 3. Occupation | Teresa Brockie, Assistant Professor Ellie Decker, Senior Research Program Coordinator Adriann Ricker, Research Associate  Theresa Aguilar, Graduate Student  Lawrence Wetsit, Cultural Research Advisor  Mary Cwik, Associate Scientist  Deborah Wilson PhD candidate, research associate  Allison Barlow, Senior Scientist Emily Haroz, Associate Scientist | N/A |
| 4. Gender | This information was not collected. | N/A |
| 5. Experience and training | Teresa Brockie has worked with Fort Peck Tribes since 2010 and has expertise in suicide and trauma research in Native American populations.  Ellie Decker received formal qualitative research training in her master's degree program and has expertise with design, coding and analysis of qualitative research. Ms. Decker also completed training on working with Native American populations.  Adriann Ricker is a member of the Fort Peck Tribes and has experience with the design, coding and analysis of qualitative research on the Fort Peck Reservation on a variety of public health topics. Theresa Aguilar has experience with qualitative data collection. Dr. Aguilar also received training on data coding from Ms. Decker and Ricker.  Lawrence Wetsit is a cultural leader and advisor and a member of Fort Peck Tribes. He is an experienced vacillator and has worked with multiple research projects.  Mary Cwik has expertise in suicide, substance use, and trauma, with a focus on preventing Native American mental health disparities.  Deborah Wilson received formal qualitative research training in her master's degree and PhD program and has expertise with design, coding and analysis of qualitative research. Ms. Wilson also completed training on working with Native American populations and is conducting her dissertation research on the Fort Peck Reservation.  Allison Barlow has expertise in behavioral and mental health interventions for Native American populations. Dr. Barlow's expertise includes suicide, depression and substance use prevention in Native American populations. Emily Haroz has expertise in mental health services for underserved populations, including Native American populations. Dr. Haroz's expertise focuses on implementation science and mental health interventions.  All researchers have qualitative research experience and/or training. | N/A |
| *Relationship with participants* | | |
| 6. Relationship established | Teresa Brockie established a research partnership with Fort Peck Tribes in 2010 and has continued to work in partnership since that time. Adriann Ricker and Lawrence Wetsit are members of Fort Peck Tribes. | 7 |
| 7. Participant knowledge of the interviewer | Prior to the start of data collection activities participants were presented with an overview of the project. Some participants had also attended a presentation on the Celebrating Life intervention from program staff and had prior knowledge of the program. Many participants were also familiar with previous work from the study team. Adriann Ricker and Lawrence Wetsit reside on the Fort Peck Reservation and are members of Fort Peck Tribes. | 10 |
| 8. Interviewer characteristics | Teresa Brockie, Adriann Ricker and Lawrence Wetsit have existing relationships and research experiences with Fort Peck Tribes. Mary Cwik and Allison Barlow have expertise in the Celebrating Life program. All researchers were familiar with the Celebrating Life program and challenges related to suicide on the Fort Peck Reservation. | N/A |
| **Domain 2: Study Design** | | |
| *Theoretical Framework* | | |
| 9. Methodological orientation and Theory | Framework analysis was utilized, which is a form of thematic analysis. | 16 |
| *Participant Selection* | | |
| 10. Sampling | Purposive sampling was utilized. | 11 |
| 11. Method of approach | Tribal Advisory Board (TAB) members were approached for participation via email. Adriann Ricker and Lawrence Wetsit approached additional FGD participants by phone and email and flyers. | 11 |
| 12. Sample size | Thirteen participants joined the study. | 11 |
| 13. Non-participation | One participant left the study early. No one refused to participate in the study. | N/A |
| *Setting* | | |
| 14. Setting of data collection | Form adaptation, round table discussions for asset mapping, and FGD 1 were collected at the Fort Peck Community College in Poplar, MT. FGD 2 was collected at the Fort Peck Reservation Tribal Headquarters in Poplar, MT. | N/A |
| 15. Presence of non-participants | No. | N/A |
| 16. Description of sample | In order to be included in the study participants needed to currently or previously work in systems of care for individuals with suicide risk and/or have knowledge of Assiniboine and Sioux culture. Participant demographic information was not collected. | 11, 15 |
| *Data Collection* | | |
| 17. Interview guide | Interview guides, forms and community asset mapping questions are available upon request. These items were not pilot tested; however, they were reviewed by all authors before use and adapted from existing measures. | N/A |
| 18. Repeat interviews | Two FGDs were conducted, using the same interview guide. All TAB member participants answered the same written asset mapping questions. No participants completed more than one FGD. | N/A |
| 19. Audio/visual recording | All data collection activities were audio recorded and transcribed. | 10, 14 |
| 20. Field notes | Field notes were taken during and after the FGDs and roundtable discussions for asset mapping by Ellie Decker or Deborah Wilson. | 15 |
| 21. Duration | FGDs were approximately one hour long. Form adaptation and community asset mapping were completed in one day, over a period of approximately 6 hours. | N/A |
| 22. Data saturation | Not discussed. | N/A |
| 23. Transcripts returned | Transcripts were not reviewed by participants. Transcripts were reviewed by study team members who had participated in data collection activities. | N/A |
| **Domain 3: Analysis and Findings** | | |
| *Data Analysis* | | |
| 24. Number of data coders | Two researchers coded data separately, and then met to review discrepancies and come to consensus on final codes. | 15 |
| 25. Description of the coding tree | The coding framework is described. Transcripts were coded by the Program Sustainability Assessment Tool (PSAT) themes. | 15-16 |
| 26. Derivation of themes | Themes were identified in advance using the PSAT for FGDs | 17-21 |
| 27. Software | Microsoft Excel was used for data coding. | 15 |
| 28. Participant checking | Participants did not provide feedback on the findings described. | N/A |
| *Reporting* | | |
| 29. Quotations presented | Yes, participants written responses to community asset mapping questions are included. FGD number identifies participant quotes. | 17-21 |
| 30. Data and findings consistent | Yes, data noted in results was consistent with overall findings noted in conclusion. | 23-24 |
| 31. Clarity of major themes | Major themes were discussed in the discussion and conclusion. | 21-24 |
| 32. Clarity of minor themes | Not discussed. | N/A |
